# The efficacy and stability of an information and communication technology-based centralized monitoring system of adherence to immunosuppressive medication in kidney transplant recipients: study protocol for a randomized controlled trial

**DOI:** 10.1186/s13063-017-2221-z

**Published:** 2017-10-16

**Authors:** Hee-Yeon Jung, Sook Jin Seong, Ji-Young Choi, Jang-Hee Cho, Sun-Hee Park, Chan-Duck Kim, Young-Ran Yoon, Hyung-Kee Kim, Seung Huh, Se-Hee Yoon, Jong Soo Lee, Yong-Lim Kim

**Affiliations:** 1 Department of Internal Medicine, Kyungpook National University School of Medicine, Daegu, South Korea; 2 Department of Biomedical Science and Clinical Trial Center, Kyungpook National University Graduate School and Hospital, Daegu, South Korea; 3 Department of Surgery, Kyungpook National University School of Medicine, Daegu, South Korea; 4 Department of Internal Medicine, Konyang University College of Medicine, Daejeon, South Korea; 5 Department of Internal Medicine, University of Ulsan College of Medicine, Ulsan, South Korea; 6 Division of Nephrology, Department of Internal Medicine, Kyungpook National University Hospital, Daegu, 41944 South Korea

**Keywords:** Information and communication technology, Adherence, Kidney transplantation

## Abstract

**Background:**

Immunosuppression non-adherence in kidney transplant recipients (KTRs) not only increases the risk of medical intervention due to acute rejection and graft loss but burdens the socioeconomic system in the form of increased healthcare costs. An aggressive preemptive effort by healthcare professionals, geared to ensure adherence to immunosuppressants in KTRs, is significant and imperative.

**Methods/design:**

This study was designed as a prospective, open-label, multicenter, randomized controlled study aimed at evaluating the efficacy and stability of an information and communication technology (ICT)-based centralized monitoring system in boosting medication adherence in KTRs. One hundred fourteen KTRs registered throughout the year 2017 to 2018 are randomized into either the ICT-based centralized home monitoring system or to ambulatory follow-up. The planned follow-up duration is 6 months. The ICT-based centralized home monitoring system described consists of a smart pill box equipped with personal identification system, a home monitoring system, an electronic Case Report Form (eCRF) system, and a comprehensive clinical trial management system (CTMS). It alerts both patients and medical staff with texts and pill box alarms if there is a dosage/dosing time error or a missed dose. Medication adherence and transplant outcomes for the follow-up period are compared between the two groups, while patient satisfaction as well as the stability and cost-effectiveness of the ICT-based monitoring system are to be evaluated.

**Discussion:**

This on-going study is expected to determine if consistent use of the ICT-based centralized monitoring system described could maximize mediation adherence and subsequently enhance transplant outcomes in KTRs. Further, it would lay the foundation for successful implementation of this ICT-based monitoring system for effective management of medication adherence in KTRs.

**Trials registration:**

ClinicalTrials.gov, Identifier: NCT03136588. Registered on 20 April 2017.

**Electronic supplementary material:**

The online version of this article (doi:10.1186/s13063-017-2221-z) contains supplementary material, which is available to authorized users.

## Background

Regular administration of immunosuppressants is critical in kidney transplant recipients (KTRs) to reduce the risk of acute rejection and enhance graft survival. A previous study has reported that the major cause of graft loss is antibody-mediated rejection which was shown to be associated with impaired adherence to immunosuppressants [1]. Non-adherence is not only medically significant, being responsible for acute rejection [2, 3] and graft loss [4], but also has wide socioeconomic implications resulting from increased healthcare costs [5] for additional hospitalization, acute rejection treatment, and re-dialysis. Given the multifaceted risk associated with immunosuppression non-adherence in KTRs, aggressive intervention by healthcare professionals to ensure adherence is imperative.

Conventional ways of assessing drug adherence include blood drug level measurement, pill counts, patient interview/use of questionnaires, and electronic monitoring. Blood drug concentration provides direct evidence, but is rarely useful due to inter-individual variability in pharmacodynamics [6]. There is also risk of errors due to the white-coat compliance where drug adherence improves abruptly for the 5 days immediately preceding a physician visit [7]. Pill count is even less reliable, barely providing information on dosing time compliance. While easy to implement, patient interview/questionnaires also pose reliability issues. Electronic monitoring has been regarded as the standard assessment of adherence [8]. It keeps records of the date/time when patients open the pill box, and the computer-connected data is downloadable and analyzable. Though the simple date/time data does not really ensure whether medication is actually taken, electronic monitoring is still considered the most reliable [9]. Henriksson et al. have conducted a study where the date/time record of immunosuppression administration in KTRs was transmitted via web-based reporting tools. Subjects were randomized to the electronic adherence monitoring system or the control system. The prospective study with 1-year follow-up has demonstrated a greater adherence with a lower rejection rate in the electronic monitoring group than in the control group [10]. In a more recent randomized clinical trial, Reese et al. have investigated KTRs who had been given pill bottles to demonstrate that the greatest adherence was achieved in those who received provider notification together with customized reminders [11].

The present clinical trial aims to investigate efficacy and stability of continuous adherence management in KTRs by means of an information and communication technology (ICT)-based centralized clinical trial monitoring system.

## Methods/design

### Study objectives

Assuming unintended forgetfulness of KTRs to take immunosuppressants oftentimes, the study seeks to identify how continuous adherence management with the ICT-based centralized clinical trial monitoring system affects drug adherence and how improved adherence is associated with therapeutic drug concentrations as well as immunological and clinical outcomes. It also aims to determine patient satisfaction with the ICT-based centralized monitoring system as well as its stability and cost-effectiveness.

### Study design

**Fig. 1:**
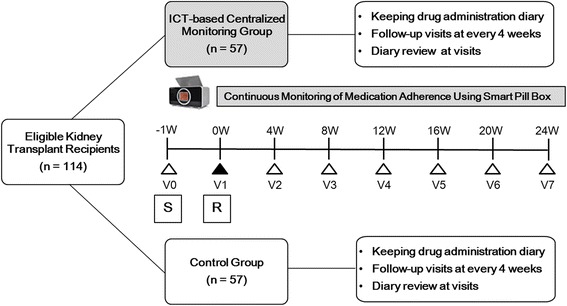
ICT-based study design. *ICT* information and communication technology, *R* randomization, *S* screening, *W* week

This study has a multicenter, open-label, prospective, randomized controlled trial (RCT) design. One hundred fourteen KTRs who fill out the Informed Consent Form are registered and randomized 1:1 into the ICT-based centralized clinical trial monitoring group (*n* = 57) or the ambulatory follow-up group (*n* = 57). The planned follow-up duration is 6 months (Fig. 1). The ICT-based centralized clinical trial monitoring group is given a smart pill box equipped with a personal identification system. Fingerprint registration is required in advance, so that it would be used for authentication before each use of the smart pill box later. The adherence-related information obtained from the pill box is saved, monitored, and sent out via a home monitoring system. Of the home monitoring system data, those necessary for the clinical trial are extracted and incorporated into the electronic Case Report Form (eCRF) system. All data is consolidated and managed within the comprehensive clinical trial management system (CTMS). In the ICT-based, centralized clinical trial monitoring group, feedback is sent to both patients and medical staff in the form of texts and pill box alarms if there is a dosage/dosing time error or a missed dose.

**Fig. 2:**
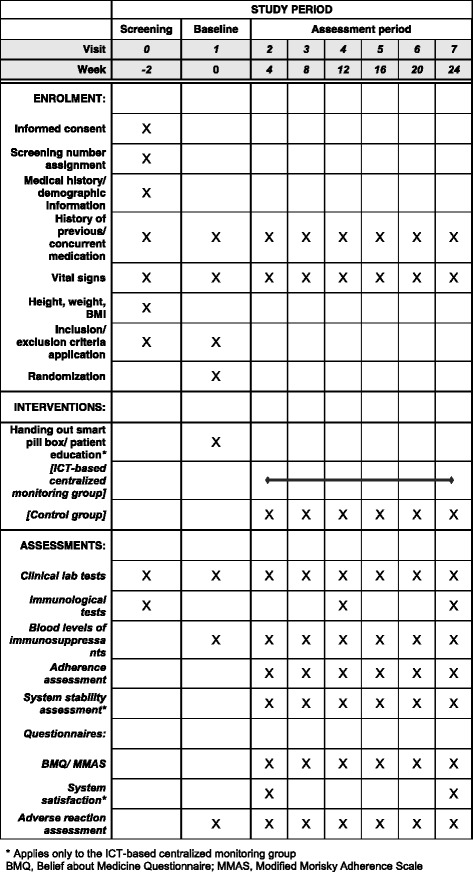
Schedule of enrollment, intervention, and assessment

Both groups are to make six office visits after randomization at 4, 8, 12, 16, 20, and 24 weeks. Each visit requires measurement of blood drug level, creatinine level, and estimated glomerular filtration rate (eGFR). Serum BK virus is assessed at 12 weeks, and panel reactive antibody (PRA) at 24 weeks. Both groups keep a drug administration diary that specifies date, whether a dose is taken or not, dosing time, and dosage. At each visit, subjects go over the diary with investigators and fill out a questionnaire using the Modified Morisky Adherence Scale [12]. The ICT-based centralized clinical trial monitoring group completes a patient Satisfaction Questionnaire developed by the ICT Clinical Trial Support Center at 4 and 24 weeks. The system Satisfaction Questionnaire can be found in Additional file 1. The protocol flow is shown in detail in the Standard Protocol Items: Recommendations for Interventional Trials (SPIRIT) figure in Fig. 2. A SPIRIT Checklist is provided in an Additional file 2.

### Sample size

We hypothesized that the ICT-based centralized monitoring group would be superior to the control group in terms of dose-taking compliance. A one-tailed test with a 0.05 significance level and 80% power provided a size of 51 in each group. With an estimated 10% dropout, the final sample size of 57 was determined (a total of 114 subjects, 57 in each group).

### Study participants

The inclusion criteria are:

1. Patients aged 8 years or older
2. At least 1 month lapsing from kidney transplantation
3. Stable renal function maintained after kidney transplantation (eGFR ≥ 30 mL/min/1.73 m^2^)
4. History of kidney transplantation only and no other transplanted organs
5. Use of tacrolimus, mycophenolic acid, and steroids for post-transplant immunosuppression
6. Patients, with the capability and willingness to give consent to trial participation, who have signed the Informed Consent Form in compliance with due process and are capable of making office visits and taking part in the trial as required by the protocol

The exclusion criteria are;

1. Patients’ refusal of the ICT-based centralized home monitoring
2. History of treatment for acute rejection within the past 3 months
3. Active infectious disease
4. Uncorrected ischemic heart disease
5. Visual or auditory defects that could affect the use of the smart pill box
6. Fingerprint authentication of personal identity deemed impossible (e.g., adermatoglyphia)
7. Illiteracy
8. Patients who do not have phones and cannot receive text messages
9. Other reasons determined by the investigators that make participation in the clinical trial inappropriate

### Randomization

Participants who will provide written consent to participate in this clinical trial will be given numbers in the order of their Informed Consent Form submission, and will undergo screening as scheduled. The screening number is a five-digit code. The first digit is the letter S, which stands for “screening.” The second digit contains the institution code, and the last three digits are determined according to the order of consent submission. After selecting appropriate participants based on the inclusion and exclusion criteria, we will give each participant a consecutive participant identification number. The participant identification number consists of five digits. The first digit is the letter R, which stands for randomization. The second digit contains the institution code, and the last three digits are determined according to the order of consent submission. Examinees will apply the participant identification numbers to a randomization table provided by a third-party statistician, and will conduct the medication adherence monitoring method.

### The ICT-based centralized clinical trial monitoring system: components and information to be collected

**Fig. 3:**
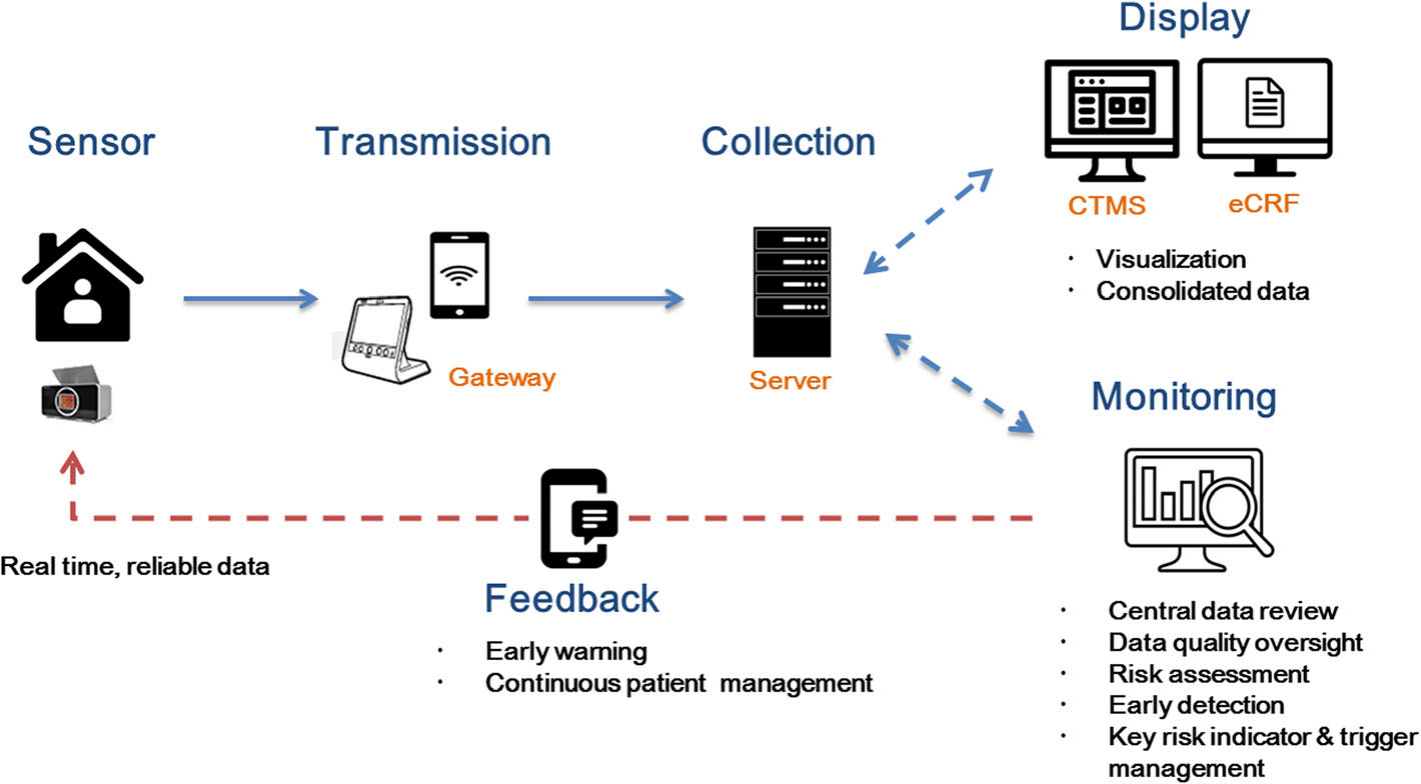
ICT-based centralized clinical trial monitoring system. *CTMS* clinical trial monitoring system, *eCRF* electronic Case Report Form, *ICT* information and communication technology

The ICT-based centralized clinical trial monitoring system consists of (1) a personal identification system, (2) a smart pill box to obtain drug adherence data, (3) a home monitoring system to save, monitor, and transmit the received data, (4) the eCRF system that selects and prepares from the home monitoring system that data necessary for the clinical trial, and (5) the comprehensive CTMS that consolidates and manages all relevant data (Fig. 3). The information obtained via the smart pill box includes whether a dose is taken or not, dosage and remaining pill counts, dosing time, and alarm times and frequency.

### Smart pill box

Information measured by smart pill boxes:

1. Medication use: the system checks whether or not the patient has ingested medications according to the clinical trial protocol and a schedule prescribed by the researchers
2. Doses of medications ingested and doses of medications remaining: the system checks the doses of medications ingested and the doses of medications remaining
3. Dosing time: the system records the actual time at which the participant ingested medications
4. Alarm time: the system records alarm times and the number of alarms set according to the patent’s medication usage

Device details:

1. Product license number: N/A
2. Product name: smart pill box
3. Model name: smart pill box
4. Manufacturer: Jeyun Medical Co.
5. Pharmacokinetics and properties:
  - Contains drugs in various forms including powder paper, capsule, and bottle
  - An automatic multi-media alarm feature that sets off the alarm according to the drug administration schedule, autonomic storage feature, and medication counseling feature
  - A sensor that measures the amount of leftover medications and a feature that monitors drugs that are placed inside or taken out of the smart pill box
  - Medication use instructions for when patients are outside. Patients can check the amount of remaining medications inside their smart pill boxes to the smallest unit
  - An alarm for missed dose, misuse, or overuse of medications
  - An electronic switch on the storage space, and emergency switching
  - Automatic formation of a database of prescribed drug administration schedules
  - Remote, real-time monitoring and control of the functional and technical conditions of the smart pill box
  - A data transfer feature (provides interfaces for WPAN, LAN, WLAN, and WAN modems)—Bluetooth, Ethernet, WiFi, 3G/4G modem interface, etc.
6. Shape/structure and size
  - Power: DC 12 V/3 A or less
  - Overall size (L–W–H): 289 × 141 × 136 (mm)/storage size (L–W–H): 206 × 105 × 74 (mm)
  - Maximum capacity: 1.0 kg
  - Display: TFT Touch LCD 3.5 inch, 320 × 240
  - Total weight: < 1.57 kg
  - Operating temperature: 0–70 °C
  - Main processor: ARM7 900 MHz Quad-core, 1 GB RAM
  - Internal memory: 8 GB, network: 10/100 Mbps Ethernet-supported, USB 2.0 4 port-supported
  - Main OS (operating system): Debian, automatic firmware upgrade supported, GUI (Graphical User Interface)-based operating system

### Home monitoring system

The home monitoring system will accumulate data about medication adherence measured from the smart pill boxes for each participant registered through the integrated management system, and will send them to the eCRF according to a defined transfer format. Moreover, when any of the situations defined in the clinical trial protocol arise, a defined feedback is sent to the patient and medical staff. The process of data transfer with the home monitoring system is summarized below;

1. The home monitoring system receives user information (personal information, screening number, etc.) from the integrated management system
2. The home monitoring system/integrated management system transfers information necessary for setting the pill box to the drug administration management platform implemented on the CTMS
3. The drug administration management platform sends information necessary for the pill box to set the drug administration information
4. Results of drug administration are sent to the home monitoring server system
5. The drug administration management platform on the home monitoring system/CTMS sends the user’s drug administration information through the home monitoring server system
6. The home monitoring server system sends the information to the eCRF
7. Feedbacks are sent to the patient’s cell phone

### The ICT-based centralized clinical trial monitoring system: feedback

In the ICT-based centralized monitoring group, both subjects and medical staff receive feedbacks regarding a missed dose, misuse, and overuse of the medication in the form of a text message alarm. In case of a missed immunosuppressant dose, the first violation generates a feedback within 1 h at the break of the ± 3-h range from the fixed dosing time. Up to two additional texts are sent at an interval of 30 min if the dose is still not taken after the feedback. For any discrepancy between the dosage taken and the dosage prescribed, a feedback is sent within 1 h from the moment of recognition. Similarly, if a dose is taken outside of the allowed ± 3-h dosing time range, a feedback is sent within 1 h of recognition. To minimize reminder fatigue, we limited additional alarms after the first feedback to two times. Any additional alarms after the two reminders will be sent to the patient’s family. Medication use information of KTRs is sent to the home monitoring system and the eCRF system in real time to allow the medical staff to check on the patients’ medication use, dosing time, and dosage. Through the eCRF system, medical staff can gain insights into all patients’ daily history of medication use, and all patients’ or a single patient’s monthly history of medication use.

### Control group

Participants in the control group will also record their daily medication use, undergo an adherence evaluation at every visit, undergo clinical laboratory and immunological tests, and have their blood levels of immunosuppressants measured.

### Questionnaires

Participants in both groups will complete the Modified Morisky Adherence Scale [13] and the Belief about Medicine Questionnaire—general (BMQ-G) [14]. We aim to investigate the patients’ level of trust on their prescriptions and their perception of medication use.

### Primary outcomes

The primary outcome in this trial is adherence to medication. Both in the ICT-based centralized clinical trial monitoring group and the control, pill counts are done at 4, 8, 12, 16, 20, and 24 weeks of follow-up. Multiple items of adherence – dose-taking compliance, dose-frequency compliance, dose-interval compliance, and drug holidays – are assessed based on the smart pill box data in the ICT-based centralized monitoring group and the drug administration diary in the ambulatory follow-up group, respectively. In the ICT-based centralized monitoring group, three types of immunosuppressants—tacrolimus, mycophenolic acid, and steroids—are placed in a single bag, which is then placed inside a smart pill box. The patient’s medication usage is assessed based on the perceived weight of the leftover medications in the bag. On the basis of this weight information, we can identify the type and dose of medications that the patient has ingested, and calculate the adherence. Each item of adherence is listed below [15]:

1. Dose-taking compliance = the number of pills taken over a certain time period/the number of pills prescribed over the same period × 100%
2. Dose-frequency compliance = the number of days of correct daily dosing frequency over a certain time period/the number of days over the same period × 100%
3. Dose-interval compliance: the number of correct dosing interval over a certain time period/the number of days over the same period × 100%.
  A correct dosing interval is defined with a ± 25% margin (in this trial, medication is taken twice a day, with a dosing interval of 12 h. Allowed dosing interval is thus ranged from 9 to 15 h)
4. Drug holidays = the number of days without dose taking/the number of days over the same period × 100%
5. Medication possession ratio = the sum of the days’ supply for all fills of a given drug in a particular time period/the number of days in the time period × 100%

### Secondary outcomes

The secondary outcomes in this trial include blood drug levels, immunological outcome, clinical outcome, patient satisfaction with the system, and the system stability and cost-effectiveness. The trough levels of tacrolimus and mycophenolic acid, creatinine level, and eGFR are measured at 4, 8, 12, 16, 20, and 24 weeks of follow-up. PRA is assessed at baseline and 24 weeks, and serum BK virus at 12 weeks. For clinical outcome evaluation, biopsy-proven acute rejection (BPAR) and graft loss developing during the follow-up are monitored. The patient Satisfaction Questionnaire is filled out at 4 and 24 weeks in the ICT-based centralized clinical trial monitoring group in order to assess how well the patients are satisfied with the system-enabled adherence management. The malfunction rate of the ICT-based centralized monitoring system is checked as well as any discrepancy between the smart pill box record and the patient-kept drug administration diary. Information about patients’ medication use sent to the ICT-based monitoring system is compared with the daily records of medication use written by the patients (type of medications used, number of pills ingested, and time of ingestion) to assess how closely they match. Stability is measured by using the following equation:

$$ \frac{\mathrm{Number}\  \mathrm{of}\  \mathrm{pills}\  \mathrm{taken}\  \mathrm{over}\ \mathrm{a}\ \mathrm{certain}\  \mathrm{period}\  \mathrm{according}\  \mathrm{to}\  \mathrm{the}\ \mathrm{ICT}\hbox{-} \mathrm{based}\  \mathrm{centralized}\  \mathrm{monitoring}\  \mathrm{system}}{\mathrm{Number}\  \mathrm{of}\  \mathrm{pills}\  \mathrm{prescribed}\  \mathrm{over}\  \mathrm{the}\  \mathrm{sampling}\  \mathrm{period}\  \mathrm{according}\  \mathrm{to}\  \mathrm{the}\  \mathrm{daily}\  \mathrm{record}\  \mathrm{of}\  \mathrm{medication}\ \mathrm{use}}\times 100\%. $$

Cost-effectiveness evaluation parameters include installation of the ICT-based centralized monitoring system, additional hospitalization due to non-adherence, ambulatory tests, and trips for hospital visits. The expenses of the ICT-based centralized monitoring system will consist of the price of the pill box itself, and the costs for transferring medication use information to the home monitoring system and the eCRF system. In cases in which patients develop acute adverse reactions due to irregular medication use and are hospitalized, receipts of the hospitalization fee, outpatient clinic visit fees, and transportation fees will be collected. We will compare differences in the ICT-based centralized monitoring system expenses, additional hospitalization fees, outpatient clinic examination fees, and transportation fees between the two groups.

### Process evaluation

The Reach, Effectiveness, Adoption, Implementation, and Maintenance (RE-AIM) framework [16] will be used in order to evaluate translatability and feasibility of ICT-based centralized monitoring system. Reach is defined as the absolute number and participation rate of eligible KTRs who participate in the ICT-based RCT. Effectiveness is defined as the improvement in adherence to immunosuppressive medication in KTRs. Adoption is defined as the absolute number, proportion, and representativeness of hospitals that are willing to participate in the ICT-based RCT. Implementation is defined as the percentage of KTRs who follow the ICT-based centralized monitoring system as intended. Maintenance is defined as the extent to which the ICT-based centralized monitoring system became part of the standard monitoring system of adherence to immunosuppressive medication in KTRs.

### System data security

All personal medical records entered in the smart pill box, drug administration management platform, the home monitoring system, the eCRF system, and the comprehensive CTMS are securely protected by means of access control and authentication, automatic user log-off, data encryption/decryption, and system log records for data audit. All systems used in the study adopt personal identifiers (ID and passwords) to ensure that different users (administrators, investigators, and subjects) are given different levels of access to data. Access is restricted if authentication expires.

A pre-defined length of inactivity results in an automatic user log-off and session end. The security measures apply to all hardware and software that collect (generate), access, store, transmit, and process any personal medical information in the home monitoring environment. The personal data (patient data, authentication data, etc.) are encrypted/decrypted when transmitted and stored by security protocols such as SEED, IPsec, SSL, and WAP. When a user obtains access, system log records are generated to track user activities including log-in/log-off, access to data, and creation/update/deletion of data. In case of any illegal access by external intruders to the information system, or of unauthorized activity by insiders, the person in charge of clinical data management should immediately report to the system manager who, if faced with risk of critical information leakage to unauthorized parties, should notify the principal investigator.

### Discontinuation/dropout criteria

A trial subject might discontinue/drop out from the trial if deemed necessary by self or by investigators in accordance with the following criteria:

1. Inappropriate registration: violation of the inclusion/exclusion criteria
2. Loss of the transplant organ
3. Request by a subject or their legal representative for discontinuation within the trial period
4. Withdrawal of consent to trial participation by a subject
5. A subject lost to follow-up
6. Other cases in which continuation of the trial is deemed not appropriate by investigators

### Statistical analysis

For categorical data, percent (%) is used for the number of subjects in a category against the total number of subjects. Analysis of categorical data uses the chi-square test or Fisher’s exact test. For continuous data, descriptive statistics (mean and standard deviation (SD)) are given, and the *t* test and the Mann-Whitney *U* test are used for analysis of normal distribution data and non-normal distribution data, respectively. The inter-group difference in adherence to immunosuppressants is assessed with the *t* test. Summary statistics of mean, SD, median, minimum value, and maximum value are presented for measurement data at each visit. The intra-group variation is analyzed with the paired *t* test or Wilcoxon’s signed rank test. Multivariate analysis is performed to identify clinical factors associated with drug compliance. Statistical significance is obtained with a *P* value of < 0.05.

## Discussion

In KTRs, poor adherence to immunosuppressants has a direct link to graft loss which in turn results in deteriorated quality of life and increased healthcare costs both for individuals and the society as a whole. A systemic and scientific approach aimed at radically improving drug adherence is imperative. An electronic monitoring device has a proven clinical utility in checking and managing drug compliance. Its routine use in clinical practice, however, is unlikely due to the need for it to be linked to additional electronic equipments. There is no available clinical study conducted in Korea on the use of such an electronic monitoring device to manage adherence to immunosuppressants in KTRs. This study is the first of its kind in Korea to investigate how continuous use of the ICT-based centralized monitoring system could improve medication adherence in KTRs and how the improvement is associated with clinical outcome. Furthermore, the study seeks to assess the stability and cost-effectiveness of the ICT-based centralized monitoring system. It is well poised to make significant contributions down the road to maximizing graft performance and clinical outcome in KTRs.

Studies have reported on technology-based intervention for improving medication adherence among KTRs. In a recent study involving 120 KTRs, the patients were randomly subjected to adherence monitoring with customized reminders (including alarms, texts, phone calls, and/or e-mails), monitoring with customized reminders plus provider notifications (every 2 weeks, providers received notification if adherence decreased to 90% during that period), or wireless pill bottle use alone (control) to assess tacrolimus adherence [11]. The provider notification plus customized reminders group showed better medication adherence than all the other groups. Zanetti-Yabur et al. compared 21 KTRs who were using a mobile phone application to improve medication adherence with 53 control patients. Although they did not find any significant differences in medication adherence between the two groups, patients who used a mobile phone application showed higher adherence [17]. McGillicuddy et al. reported significant improvements in medication adherence after comparing a smartphone-based mobile health intervention group (*n* = 9) with a control group (*n* = 10) [18]. The use of pill boxes has been clinically shown to lower blood pressure and increase adherence to antiretroviral therapy not only in KTRs but also in patients with uncontrolled hypertension [19] and human immunodeficiency virus (HIV) infection [20].

Backed by rapid expansion in IT infrastructure, spread of the use of personal smart devices, and progress in u-Health technology, interest in the ICT-based clinical trial has been on the rise, and there indeed have been attempts at providing an ICT-based healthcare service. Around the globe, the incorporation of ICT into the clinical trial industry is in its infancy regarding developmental stage, and with little standardized system at present, there exists much room for further pioneering and development. This study is significant as it would pave the way for establishing the ICT-based centralized monitoring system necessary for effective management of drug compliance. Solid groundwork laid by this study might have broad implications for all other patient populations – recipients of other organ transplant, chronic suffers of hypertension, diabetes, or chronic kidney disease, patients with HIV infection, and tuberculosis patients, for example – for whom adherence to medication is undoubtedly critical in the course of treatment.

Despite the significance of its results, our study has the following limitations. First, we assumed that the reason for patients not taking their medications is unintended forgetfulness, and we investigated whether we could enhance adherence by providing the patients with reminder alarms from the ICT-based centralized monitoring system. However, this system does not use a camera to pictorially record the patients at the moment of medication ingestion; therefore, it cannot improve intentional non-adherence. To overcome this limitation, we will consistently educate our patients on the importance of medication adherence to reduce intentional medication non-adherence. In addition, we will use the BMQ-G to understand the reasons for patient non-adherence and find solutions to this problem. Second, even if patients ingest the correct amount of medications at the right time, there can be differences in drug level, or the secondary outcome, among the patients, owing to intra- and inter-patient variabilities [21, 22]. Therefore, it is rather difficult to use drug levels as the secondary outcome and they must be carefully interpreted.

To fully establish, expand, maintain, and effectively run an ICT-based centralized monitoring system for improving immunosuppressant adherence in KTRs in real-world health systems, it is necessary to use a standard framework such as the RE-AIM framework [16]. We plan to establish and evaluate the system via common and effective evidence-based interventions by using a standard framework.

### Trial status

The ICT-based randomized controlled clinical study was ongoing at the time of article submission in May 2017.

## Additional files

Additional file 1: Satisfaction Questionnaire – information and communication technology (ICT)-based centralized monitoring system. (DOCX 86 kb)

Additional file 2: SPIRIT 2013 Checklist: recommended items to address in a clinical trial protocol and related documents*. (DOC 123 kb)

## Abbreviations

BPAR: Biopsy-proven acute rejection
CTMS: Comprehensive clinical trial management system
eCRF: Electronic Case Report Form
eGFR: Estimated glomerular filtration rate
HIV: Human immunodeficiency virus
ICT: Information and Communication Technology
KTRs: Kidney transplant recipients
PRA: Panel reactive antibody
RCT: Randomized controlled trial
RE-AIM: Reach, Effectiveness, Adoption, Implementation, and Maintenance
