# Supplementary material for: The efficacy and stability of an information and communication technology-based centralized monitoring system of adherence to immunosuppressive medication in kidney transplant recipients: study protocol for a randomized controlled trial
Source: Trials. 2017 Oct 16;18:480. doi: 10.1186/s13063-017-2221-z (PMC5644178; doi:10.1186/s13063-017-2221-z)
Supplement: Supplementary file 1 — Satisfaction Questionnaire – information and communication technology (ICT)-based centralized monitoring system. (DOCX 86 kb) [file 13063_2017_2221_MOESM1_ESM.docx]

**Satisfaction questionnaire – information and communication technology (ICT)-based centralized monitoring system**

| Efficacy and Stability of Information and Communication Technology-based Centralized Monitoring System of Adherence to Immunosuppressive Medication in Kidney Transplant Recipients: A Prospective, Randomized Controlled, Multicenter Study | |
| --- | --- |
| **Research director** | **Kyungpook National University Hospital – Professor Yong-Lim Kim**  **Ulsan University Hospital – Professor Jong Soo Lee**  **Konyang University Hospital – Professor Se-Hee Yoon** |

| **Participant screening number** |  |
| --- | --- |
| **Visit number** |  |

The present data will not be used for a purpose other than for this research, and all personal information will be kept confidential. Only participants who have consented to participate in this research will be able to answer this questionnaire. Completion of this questionnaire will not entail any kinds of disadvantage. You may refuse to answer this questionnaire if you so wish. You may also withdraw from completing this questionnaire even after you have started.

**We would like to thank you for your time once again.**

201 Year        Month        Day


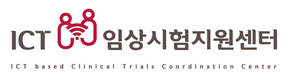


| **Directions** |
| --- |

1. A participant must complete this questionnaire on his/her own will, and his/her responses should not reflect the researchers’ opinions.
2. A participant must complete this questionnaire during a visit to the hospital and submit it to the researchers/research nurses immediately after completing it.
3. A participant must use a black ballpoint pen to write on this questionnaire and must not record his/her responses in the blank spaces of the pages.
4. A participant must provide as many details as possible concerning his/her responses and try not to leave any empty spaces for answers.
5. For confidentiality reasons, a participant’s name and the hospital registration number should not be recorded on this questionnaire.

**Ⅰ. General Information**

Circle or check an option that applies to you.

| Age | 20s | 30s | 40s | 50s | 60s or above | Sex | Male | Female |
| --- | --- | --- | --- | --- | --- | --- | --- | --- |

| Education level | Elementary school | Middle school | High school | University | Above university |
| --- | --- | --- | --- | --- | --- |

| Monthly household income (more than two members per family, full) | Less than 200 | 200–399 | 400–599 | Greater than 600 |
| --- | --- | --- | --- | --- |

| Area of residence | Large city (metropolitan city) | Small- to medium-sized city | Agricultural and fishing village | Smartphone use | Yes | No |
| --- | --- | --- | --- | --- | --- | --- |

| Frequency of searching health information (information about diseases, symptoms, medications, etc.) on the Internet or through wireless communications | Once per week | 3 times per week | Every day |
| --- | --- | --- | --- |

| Occupation |  | Please write as many details as you can. |
| --- | --- | --- |

**Ⅱ. Level of understanding about the ICT-based centralized monitoring system**

1. Have you participated in a remote medical care project or clinical trial using information and communication technology (ICT) (TV, broadcasts, computer, cell phone, Internet, big data, Internet of Things (IoT), cloud, or mobile applications)?

| ① No | ② Yes (🞎Hospital 🞎Clinic 🞎Health center/health-care center 🞎Others (________) ) |
| --- | --- |

1. How familiar are you with the ICT-based centralized monitoring system that you are currently using?

| ① Not familiar at all | ② Not familiar | ③ Neutral | ④ Familiar | ⑤ Very familiar |
| --- | --- | --- | --- | --- |

1. How helpful were the education and information sheets about the home monitoring devices in your understanding of the ICT-based centralized monitoring system?

| ① Not helpful at all | ② Not helpful | ③ Neutral | ④ Helpful | ⑤ Very helpful |
| --- | --- | --- | --- | --- |

**Ⅲ. Satisfaction with the ICT-based centralized monitoring system**

※The following questions assess your satisfaction with the ICT-based centralized monitoring system. Please choose (√) an option that closely matches your opinion.

| **ICT-based centralized monitoring system**  **= fingerprint sensor + home monitoring devices (smart pill box/ PT/INR monitor/ blood glucose meter/ ECG monitor/ spirometer)**  **+ gateway, smartphone apps + modem** |
| --- |

| Questions | Very unsatisfied | Unsatisfied | Neutral | Satisfied | Very satisfied |
| --- | --- | --- | --- | --- | --- |
| 1. Are you satisfied with the system, overall? | ① | ② | ③ | ④ | ⑤ |
| 2. Was the system convenient to use? | ① | ② | ③ | ④ | ⑤ |
| 3. Was the clinical trial using the system safe? | ① | ② | ③ | ④ | ⑤ |
| 4. Did the use of the system reduce the duration of the trips you made to participate in this clinical trial? | ① | ② | ③ | ④ | ⑤ |
| 5. Did the use of the system reduce the inconvenience associated with transportation? | ① | ② | ③ | ④ | ⑤ |
| 6. Were the researchers able to assess your conditions more accurately by using the system? | ① | ② | ③ | ④ | ⑤ |
| 7. Were the measurements obtained from the devices included in the system (fingerprint sensor, home monitoring devices, gateway/smartphone apps, modem, etc.) reliable? | ① | ② | ③ | ④ | ⑤ |
| 8. Were the aforementioned devices easy to use? | ① | ② | ③ | ④ | ⑤ |
| 9. Are you satisfied with the education on the directions and precautions for use of the aforementioned devices? | ① | ② | ③ | ④ | ⑤ |
| 10. Are you satisfied with how the researchers handled errors that arose from the aforementioned devices? | ① | ② | ③ | ④ | ⑤ |

11. Please describe the qualities of the ICT-based centralized monitoring system that you were most satisfied with.

:___________________________________________________________________________________________

12. Please describe the qualities of the ICT-based centralized monitoring system that you were most unsatisfied with.

: __________________________________________________________________________________________

13. Of the devices included in the ICT-based centralized monitoring system, which ones were you the most dissatisfied with? (Please circle your choice.)

| ① Fingerprint sensor | ② Home monitoring device (smart pill box/ PT/INR monitor/ blood glucose meter/ ECG monitor/ spirometer) | |
| --- | --- | --- |
| ③ Gateway | ④ Smartphone app | ⑤ Modem |

14. Did errors arise from any of the devices included in the ITC-based centralized monitoring system?

| ① No |  |
| --- | --- |
| ② Yes🡺 | 1. Error frequency: _________________ errors/week |
|  | 1. If yes, how did you handle the errors? |
|  | ① I did not handle them.  ② I handled them by myself.  ③ I asked my physician.  ④ I asked my nurse.  ⑤ I asked my family. |

**Ⅳ. Questions about the introduction of a clinical trial using the ICT-based centralized monitoring system**

※Please choose (√) an option that closely matches your opinion.

| Questions | Never | No | Neutral | Yes | Most certainly |
| --- | --- | --- | --- | --- | --- |
| **If the ICT-based centralized monitoring system is introduced into this clinical trial,** | | | | | |
| 1. Will you consistently participate in this clinical trial using the ICT-based centralized monitoring system? | ① | ② | ③ | ④ | ⑤ |
| 2. Will you participate in this clinical trial even if it takes place at a hospital located farther away from your home owing to the availability of the system at that location? | ① | ② | ③ | ④ | ⑤ |
| 3. Was this clinical trial using the system helpful for the management of your health? | ① | ② | ③ | ④ | ⑤ |
| 4. Do you think a clinical trial using the system will positively contribute to your quality of life? | ① | ② | ③ | ④ | ⑤ |
| 5. Would you recommend a clinical trial using the system to others? | ① | ② | ③ | ④ | ⑤ |
| 6. Do you think clinical trials using the system may lead to any losses or damage associated with personal medical information leakage? | ① | ② | ③ | ④ | ⑤ |
| 7. Do you think it will become more difficult to use medical services owing to technical issues associated with the system? | ① | ② | ③ | ④ | ⑤ |
| 8. Do you think technical issues associated with the system will give rise to medical accidents? | ① | ② | ③ | ④ | ⑤ |

9. Introducing a clinical trial using the system can lead to expenses from the use of devices that constitute the system, as well as communication charges. To what extent should participants be given financial support in this clinical trial relative to the level of financial support provided during the existing clinical trial?

| Financial support for the existing clinical trial | ICT-based monitoring system expenses |
| --- | --- |
| Examination fees + transportation fees | Examination fees + device use fees + communication fees |

1. Participants can be given less financial support than they received in the existing clinical trial.
2. Participants should be given the same level of financial support as they received in the existing clinical trial.
3. Participants should be given more financial support than they received during the existing clinical trial. 🡺 For each visit, up to ___________________ won must be provided.

**Ⅴ. If you have any opinions about the ICT-based centralized monitoring system, and a clinical trial using this system, please share them below.**

|  |
| --- |

- Thank you for completing this questionnaire. -
